# Using principles of motor control to analyze performance of human machine interfaces

**DOI:** 10.1038/s41598-023-40446-5

**Published:** 2023-08-15

**Authors:** Shriniwas Patwardhan, Keri Anne Gladhill, Wilsaan M. Joiner, Jonathon S. Schofield, Ben Seiyon Lee, Siddhartha Sikdar

**Affiliations:** 1 Department of Bioengineering, George Mason University, Fairfax, VA 22030 USA; 2 Department of Psychology, George Mason University, Fairfax, VA 22030 USA; 3 Department of Neurobiology, Physiology and Behavior, University of California, Davis, Davis, CA 95616 USA; 4 Mechanical and Aerospace Engineering Department, University of California, Davis, Davis, CA 95616 USA; 5 Department of Statistics, George Mason University, Fairfax, VA 22030 USA; 6 Center for Adaptive Systems of Brain-Body Interactions, Fairfax, VA 22030 USA

**Keywords:** Biomedical engineering, Motor control, Brain-machine interface

## Abstract

There have been significant advances in biosignal extraction techniques to drive external biomechatronic devices or to use as inputs to sophisticated human machine interfaces. The control signals are typically derived from biological signals such as myoelectric measurements made either from the surface of the skin or subcutaneously. Other biosignal sensing modalities are emerging. With improvements in sensing modalities and control algorithms, it is becoming possible to robustly control the target position of an end-effector. It remains largely unknown to what extent these improvements can lead to naturalistic human-like movement. In this paper, we sought to answer this question. We utilized a sensing paradigm called sonomyography based on continuous ultrasound imaging of forearm muscles. Unlike myoelectric control strategies which measure electrical activation and use the extracted signals to determine the velocity of an end-effector; sonomyography measures muscle deformation directly with ultrasound and uses the extracted signals to proportionally control the position of an end-effector. Previously, we showed that users were able to accurately and precisely perform a virtual target acquisition task using sonomyography. In this work, we investigate the time course of the control trajectories derived from sonomyography. We show that the time course of the sonomyography-derived trajectories that users take to reach virtual targets reflect the trajectories shown to be typical for kinematic characteristics observed in biological limbs. Specifically, during a target acquisition task, the velocity profiles followed a minimum jerk trajectory shown for point-to-point arm reaching movements, with similar time to target. In addition, the trajectories based on ultrasound imaging result in a systematic delay and scaling of peak movement velocity as the movement distance increased. We believe this is the first evaluation of similarities in control policies in coordinated movements in jointed limbs, and those based on position control signals extracted at the individual muscle level. These results have strong implications for the future development of control paradigms for assistive technologies.

## Introduction

In recent years, there have been significant advances in the control of biomechatronic devices driven by biological signals derived from the user^1–9^. Various biological signals have been used for gesture intent recognition to drive such biomechatronic devices, including electrical signals such as surface electromyography (sEMG)^10–12^, electroencephalography^13,14^, electrocorticography^15,16^, as well as mechanical signals such as mechanomyography^17^ and sonomyography^18–24^. These signal extraction techniques have found broad applications in rehabilitation engineering^25–27^. However, these techniques have their own limitations. In some of these techniques, the users have control over only a few degrees of freedom, while in others, the user can proportionally control movement velocities, one gesture at a time, and need to use an ad hoc triggering mechanism to cycle through different gestures^26,28,29^. Clinically, there continues to be a significant unmet need to improve the control of biomechatronic devices. Prosthetic users can often reliably control only a small number of degrees of freedom of advanced multiarticulated prosthetic hands^30^. Nearly all of the individuals affected by stroke, cerebral palsy, or Parkinson’s disease often fail to attain normal function and activity levels even with the help of modern rehabilitation practices and assistive devices^31^. Exoskeletons have been designed to achieve better joint function^32–34^, but effective control over the exoskeleton is a critical limiting factor^35^.

To address these limitations there is extensive ongoing work on developing sophisticated machine learning algorithms to decode motor intent from non-invasive sensing modalities, as well as research on acquiring signals with increased signal to noise ratio and better specificity. Examples of ongoing algorithmic research includes pattern recognition^36–39^, artificial neural networks^40^ and regression-based techniques^41–46^. Examples of techniques being pursued to overcome the low signal-to-noise^47,48^, and low specificity^49,50^ of conventional sEMG include subcutaneously implanted electrodes^51,52^, or specialized surgical procedures like targeted muscle reinnervation^53^ as well as emerging noninvasive sensing modalities such as sonomyography^18–24^.

We chose to evaluate a human machine interface controller based on sonomyography, an emerging control method that uses real-time dynamic ultrasound imaging of deep lying musculature to infer motor intent. While the methods proposed in this paper can be extended to any human machine interface controller, we chose to use sonomyography because of its potential to achieve high signal to noise ratio for proportional positional control. Prior work from our group^18–21^ and others^22–24^ has shown real-time ultrasound imaging of forearm muscle deformations during volitional motor action can be used to decode motor intent during proportional isotonic movement^54^. In our previous work^18^, we showed that able bodied individuals as well as persons with limb deficiency were able to accurately and precisely control a virtual cursor using sonomyography.

In this paper, we investigate whether the concept of minimum jerk trajectory can be utilized to evaluate the performance of a human machine interface controller based on sonomyography. It has been shown humans follow typical minimum jerk trajectories during a variety of tasks such as arm reaching^55–57^, catching^58^, drawing movements^59^, vertical arm movements^60^, head movements^61^, saccadic eye movements^62^, chewing^63^, and several other motor skills^64^. Showing such a dynamic behavior of the task trajectories would be valuable to assess task performance from a motor control perspective. Although this minimum jerk trajectory has been shown for multi-joint coordinated movements such as reaching tasks, these studies predominantly measure the trajectory of some kinematic displacement. However, it has not been shown to exist during target acquisition tasks where the control signals are derived from an internal measurement using a sensor that is directly tracking muscle activation (i.e., not the resulting movement of the end-effector). Our objective in this paper is to evaluate the extent control signals derived at the muscle level follow the time course of minimum jerk trajectories.

To examine the extent sonomyography-based control signals mimic those of the intact limb, we developed two experiments. In Experiment 1, subjects controlled a virtual cursor position based on the position of a hand-held manipulandum. This data was collected to characterize typical baseline multi-joint coordinated movements. In Experiment 2, the same subjects moved a virtual cursor based on the control signals derived from sonomyography of the forearm muscles during wrist flexion-extension. Our research objective was to characterize the trajectories derived from the activation of the lower level muscle groups of the forearm (Experiment 2) and determine the extent to which they reflected the trajectories derived from multi-joint coordinated movement of the upper limb (Experiment 1). We hypothesized that similar to the point-to-point reaching movements performed using a hand-held manipulandum, the trajectories resulting from sonomyography would follow a minimum jerk trajectory, resulting in a systematic scaling in peak movement velocity magnitude and delay in time for the peak to occur as the movement distance increased. Such an analysis of movement trajectories provides not only all the standard metrics such as accuracy and path length, but also valuable time-domain information regarding the dynamics of the movement.

## Methods

### Participants

The same ten able-bodied individuals (mean age: 30 ± 5 years, 5 female) were recruited for both the experiments. Eight participants reported being right-hand dominant. All experiments described in this work were approved by the George Mason University Institutional Review Board and performed in accordance with relevant guidelines and regulations. All participants provided written, informed consent prior to participating in the study, and were also compensated for their participation.

**Figure 1:**
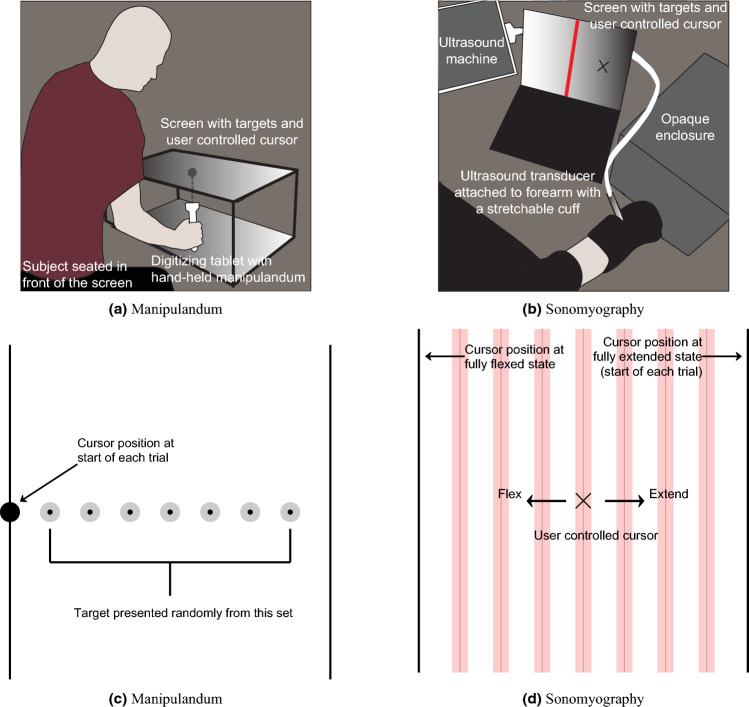
(**a**) Setup for Experiment 1. Subjects were seated in front of a digitizing tablet to capture the movement trajectories as they moved a manipulandum. A LCD monitor was placed above the tablet that showed the target location and the subjects’ manipulandum position. The subjects were asked to achieve the target positions displayed on the screen. (**b**) Setup for Experiment 2. Subjects were instrumented with an ultrasound transducer that recorded cross-sectional ultrasound images of their forearm, which resulted in a proportional signal correlating to their extent of flexion. The position of the subject-controlled cursor on the screen in front of them was driven by the proportional signal derived from sonomyography. Subjects were asked to achieve the target positions displayed on the screen. (**c**) Diagram showing the target acquisition task performed in Experiment 1. The dark circle on the left shows the starting position, while all the possible target locations are shown as faint circles on the screen. (**d**) Diagram showing the target acquisition task performed in Experiment 2. All possible targets are given by faint red lines. The ’X’ indicates the user controlled cursor. Flexing their wrist would make the cursor go left and extending their wrist would make the cursor go right (for a right handed person). It would be opposite for a left handed person. A fully extended wrist state would correspond with the cursor being at the right-most edge of the screen and a fully flexed state of the wrist would correspond with the cursor being in the left-most edge of the screen (for a right handed person).

### Experimental setup and procedure

#### Experiment 1

Participants were asked to sit upright in front of a waist-high table. They sat in front of a horizontal 27-inch LCD monitor. The chair height was adjusted for each subject so that they could comfortably perform the task and view the screen. The experimental system included a monitor, a digitizing tablet, and a Windows PC to run the experimental paradigm and collect the behavioral data. The LCD monitor was mounted horizontally in front of subjects, displaying the target locations to achieve during each trial. The monitor was 10 inches above the digitizing tablet (Intuos4, Wacom) that tracked and recorded hand position at 60 Hz. Subjects grasped a cylindrical handle with their right hand, 2.5 cm in diameter, containing the tablet stylus inside. The hand/stylus moved on the tablet below the monitor. The position of the LCD monitor obstructed the vision of the tablet and the arm movements made by subjects. This setup is referred to as the ‘manipulandum’ setup (Fig. 1a,c).

The task was designed in PsychoPy and the data was collected and stored after being de-identified, with only a subject ID number. The participants were asked to move the manipulandum such that they were within a small circle (diameter 5 cm) to the left edge of the screen. Once they arrived at this point and held their position for 3 s, a target location was shown to the right of this position on the screen and they were prompted to acquire this newly presented target (diameter 5 cm). Once they moved the manipulandum to the correct target position, and held this new target position for 3 s, they were asked to move back to the original position to the left edge of the screen. This describes one trial. Seven such targets were displayed in each block of trials, and each block was repeated 5 times. This resulted in each participant performing the task 35 times (7 targets presented 5 times each). All the subjects performed the task with their right hand, with targets being presented to the right of the user and the user always starting from the left edge of the screen. The movement trajectory taken by the subject on each trial was sampled at 60 Hz and stored in separate tables. This data was analyzed post-hoc using custom-developed scripts in MATLAB.

#### Experiment 2

Participants were asked to sit upright with their elbow below their shoulder and the forearm comfortably secured to a platform on the armrest of the chair. Participants were instrumented with a clinical ultrasound system (Terason uSmart 3200T) connected to a low-profile, high frequency, linear, 16HL7 transducer. The imaging depth was set to 4 cm and the gain was set to 60. The transducer was manually positioned on the volar aspect of the forearm (dominant arm), in order to access the deep and superficial flexor muscles of the forearm. Subjects performed the task with their dominant limb. Left handed users started with their left wrist fully extended and the cursor to the left most edge of the screen with targets being presented to the right. Right handed users started with their right wrist fully extended and the cursor to the right most edge of the screen with targets being presented to the left. The transducer was secured in a custom designed probe holder and held in place with a stretchable cuff. In order to to prevent direct observation of the wrist and hand movements, participants placed their hand in an opaque enclosure. A USB-based video grabber (DVI2USB 3.0, Epiphan Systems, Inc.) was used to transfer ultrasound image sequences in real time to a PC (Dell XPS 15 9560). The acquired image frames were processed in MATLAB (The MathWorks, Inc.) using custom-developed algorithms^18^. The participants had a computer screen in front of them where they could see a real-time plot of their derived proportional position, along with the virtual targets. This setup is referred to as the ‘sonomyography’ setup (Fig. 1b,d).

Participants underwent a training procedure in which they performed repeated wrist extension and flexion. These movements were timed to a metronome such that the participant was cued to transition from full extension to full flexion within one second, hold the position for one second, return to full extension within one second, and hold the position for one second. This process was repeated five times. The ultrasound images corresponding with the full extension and flexion phases were averaged into a single representative image each, and added to a training database with a corresponding ‘extension’ or ‘flexion’ label. This formed the reference data set. The training captured the users’ flexion/extension end-states, and mapped those two end-states onto the two edges of the screen, such that all partial states would have a corresponding position on the screen for the cursor. Our training method has been described in detail in our previous work^18^. We only asked the users to move as accurately as possible, and told them that they had 10 s to do this, before the trial timed out. We asked the users to perform comfortable levels of flexion/extension during training, so that they would be able to perform these motions repeatedly.

During the actual trials, cross-sectional ultrasound images of the subject’s forearm were compared to this reference data set to derive a proportional signal as described in our prior work^18^. This derived proportional signal was sent to the computer, where the participant controlled an on-screen cursor that could move horizontally in proportion to the degree of muscle activation in the forearm (i.e., the cursor moved left in response to wrist flexion and right in response to wrist extension for right-handed subjects and opposite for left-handed subjects). The objective was to reach a vertical target line as accurately as possible before the trial ended, and retain the cursor at the target line until the trial ended. The interface presented a target position at random from a set of seven predefined, equidistant positions, which were identical to the target distances presented in Experiment 1. The target remained at each position for 10 s and then moved to the next position until all seven target points were presented. For each target position, the participant was prompted, via an automated audio cue, to move the cursor to rest position before the task began. After the seven targets were presented, the participant would rest for one minute and then repeat the task. They completed six blocks, with the first block for practice, and the following five blocks were used for analysis (7 targets, presented 5 times each).

### Computation of movement parameters

For both the experiments, movement trajectories were recorded along with the associated timestamp and imported into MATLAB. No trials were discarded. The following metrics were computed.

#### Movement distance

Seven targets were presented at $12.5\%$, $25\%$, $37.5\%$, $50\%$, $62.5\%$, $75\%$, and $87.5\%$ of screen width. For Experiment 1, the width of the manipulandum tablet was the same as the workspace shown on the 27 inch LCD screen above it (see Fig. 1a). For Experiment 2, a 27 inch computer monitor showed the cursor and target locations. The distance between the start point (always at 0% of screen width) and the target was the movement distance. The movement of the cursor in Experiment 2 was a virtual movement proportional to the range of motion, but scaled to the size of the screen, meaning that a fully extended state of the hand made the cursor go one extreme edge of the screen whereas a fully flexed state made it go to the other extreme. For example, 50% movement distance would require moving the cursor 13.5 inches on the screen. In this work, all movement distances and movement velocities have been expressed in terms of percentage of screen width. The target in Experiment 1 was a filled grey circle with a fixed radius, and the users were asked to reach a black dot at the center of the target as accurately as possible. In Experiment 2, the target was a filled red vertical band with a fixed width, and the users were asked to reach a one-pixel wide black line at the center of the target as accurately as possible.

#### Trial start and end point

The start point of each trajectory was set to the first sample where the movement velocity was positive. This was defined as the movement onset. The trial was marked as finished when the user-driven cursor was greater than $95\%$ of the target for the first time.

#### Time to target

The time taken by the cursor to move from the start point to the end point was defined as the time to target for that trial. For every movement distance, we computed an average time to target and used it to compute the minimum jerk trajectory for that target.

#### Minimum jerk trajectory

The minimum jerk trajectory (MJT) minimizes the acceleration changes over the duration of the movement. This has been shown to be followed extensively throughout a wide variety of human movements. The MJT is represented as

1 $$\begin{aligned} MJT = \int _{t_s}^{t_f} ||\dddot{x}(t)||^2 dt \end{aligned}$$

The position and velocity profiles resulting from Eq. (1) as functions of time (t) are given^65^ by

2 $$\begin{aligned} x(t)=\, & {} x_0 + (x_f - x_0)(10\uptau ^3 - 15\uptau ^4 + 6\uptau ^5) \end{aligned}$$

3 $$\begin{aligned} v(t)=\, & {} \frac{1}{d}(x_f - x_0)(30\uptau ^2 - 60\uptau ^3 + 30\uptau ^4) \end{aligned}$$

where,

$$\begin{aligned} x(t)&= \text {Position of cursor at time t}\\ v(t)&= \text {Velocity of cursor at time t}\\ t_s&= \text {Start time}\\ t_f&= \text {Finish time}\\ d&= \text {Time to target, } t_f - t_s\\ x_0&= \text {Starting position}\\ x_f&= \text {Target position}\\ \uptau&= \frac{t}{d} \end{aligned}$$

#### Position error

The average root mean square error between the cursor position and the minimum jerk trajectory over the duration of the movement was defined as the position error.

#### Path Efficiency

Path efficiency is the ratio of actual path length to the ideal path length. The actual path length was calculated by measuring the total distance traversed by the user to settle at the target. The ideal path length was defined as the distance between the starting point and the center of the target. For example, if for a given movement distance, the user-cursor trajectory started at 0 and traversed through points A-B-C to finally reach the target, then the path length would be 0A+AB+BC.

### Statistical analysis

We employed a two-stage approach to assess the effects of the interactions between control modality and movement distance. We utilized the Scheirer–Ray–Hare (SRH) test, an extension of the Kruskal-Wallis test to experimental designs with multiple factors^66,67^. Similarly, the SRH test is a non-parametric analogue to multiple-factor analysis of variance (ANOVA).

In the first stage, we ran four total SRH tests corresponding to each response variable - peak velocity achieved during the trial, time taken to achieve the target, position error between target and cursor, and the time to velocity peak. The factors included control modality (Manipulandum and Sonomyography), movement distance (12.5%, 25%, 37.5%, 50%, 62.5%, 75%, and 87.5% of screen width), and an interaction effect between control modality and movement distance. The type of analysis performed in the second stage depended on whether the interaction term was significant in the first stage.

In the second stage, for response variables that did not have a significant effect with respect to the interaction term, we performed an updated set of SRH tests omitting the interaction term. For the response variables that had a significant interaction effect between control modality and movement distance, we performed two separate Kruskall–Wallis (KW) tests for subsets of the trials. The first subset consists of trials performed at the seven movement distances corresponding to the manipulandum modality, and the second subset includes the remaining trials performed at the seven movement distances obtained via sonomyography. After subsetting, note that there is just a single factor (movement distance) for each KW test. We elected to fix control modality for the Kruskal Wallis tests to minimize the total number of tests. However, one may choose to fix movement distance in future studies.

Two Brown-Forsythe tests were performed to test the effect of control modality on the variance of time to target and peak velocity across modalities. Two Brown-Forsythe tests were also performed to test the effect of movement distance on variance in path efficiency for the same control modality. A statistical significance level of *p* $=$ 0.05 was used throughout this work.

**Figure 2:**
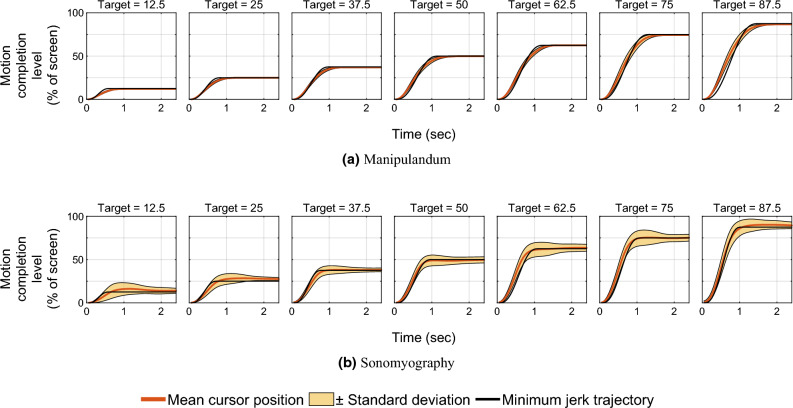
Position traces versus time. The red line shows the mean position trace across all subjects, and the shaded yellow region shows one standard deviation. The horizontal axis represents time (seconds) from movement onset at time zero, and the vertical axis represents the distance to the target as a percentage of the workspace. The black line represents the minimum jerk trajectory based on the time to reach the target.

**Figure 3:**
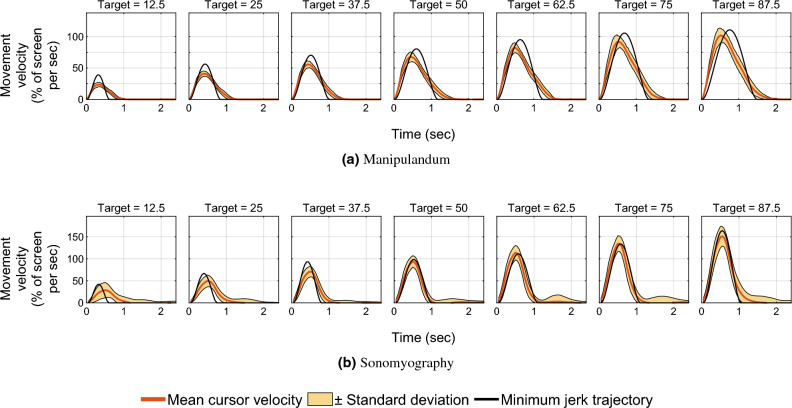
Velocity traces versus time. The red line shows the mean velocity trace across all subjects, and the shaded yellow region shows one standard deviation. The horizontal axis represents time (seconds) from movement onset at time zero, and the vertical axis represents the movement velocity as a percentage of the screen covered per second. The black line represents the minimum jerk trajectory based on the time to reach the target.

**Figure 4:**
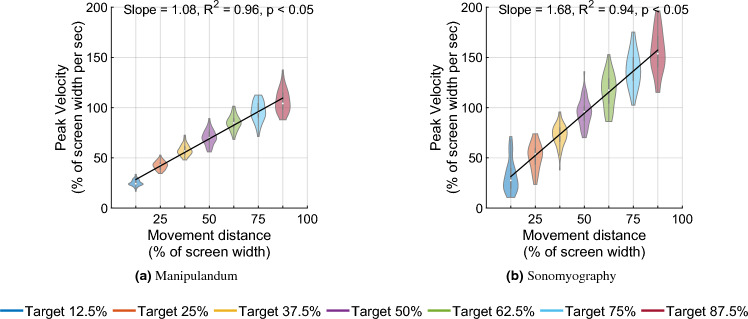
Peak velocity versus movement distance. Each violin shows the distribution of peak velocities for all trials plotted against movement distance for those trials. As the movement amplitude increased, the subjects proportionally increased the peak movement velocity.

**Figure 5:**
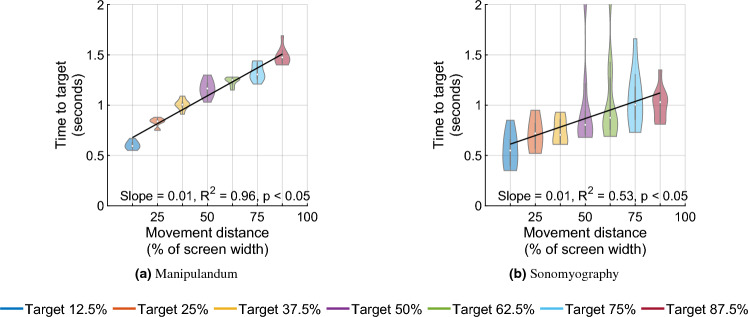
Time to target in seconds. The time take by subjects to acquire a specific target increased as the movement distance to that target increased.

## Results

In Experiment 1, we quantified the movement parameters while subjects made reaching movements of different amplitudes. Subjects moved a virtual cursor to one of seven different target positions. The cursor location reflected the location of the manipulandum moved by the subject. We analyzed performance by examining the trajectories of the cursor for each movement distance. As shown in prior literature^55,68,69^, the trajectories of the movement had a single velocity peak at approximately their mid-point, and a bell-shaped velocity profile (Figs. 2a and 3a).

In Experiment 2, we characterized performance when the cursor was driven using sonomyography. The movement distances were the same as those used in the arm reaching task with the manipulandum. In this case subjects moved the cursor to the required target by flexing their forearm muscles to the appropriate level. As above, we analyzed performance by examining the trajectories of the cursor for each movement distance. As was the case for the reaching movements, the trajectories taken by the subjects had a single velocity peak at approximately their mid-point, and a bell-shaped velocity profile (Figs. 2b and 3b).

In the first stage, a two-way Scheirer–Ray–Hare test was performed to test the effect of the control modality and the movement distance on the peak velocity achieved during a trial. The peak velocity increased significantly with respect to the movement distance across both control modalities (Fig. 4, *p* < 0.05, degrees of freedom = 6, H = 578.13). The peak velocity also increased significantly with respect to control modality (*p* < 0.05, degrees of freedom = 1, H = 57.27, higher for Experiment 2 than Experiment 1), but did not change significantly with the interaction term (*p* $=$ 0.08, degrees of freedom 6, H = 11.12). In the second stage, an updated SRH test omitting the interaction term showed that the peak velocity increased significantly with respect to movement distance (*p* < 0.05) and increased significantly with respect to control modality (*p* < 0.05, higher for Experiment 2 than Experiment 1). A linear regression showed that the peak velocity increased linearly with respect to movement distance for Experiment 1 (slope = 1.08, $R^2=0.96$) as well as Experiment 2 (slope = 1.68, $R^2=0.94$).

In the first stage, a two-way Scheirer–Ray–Hare test was also performed to test the effect of the control modality and the movement distance on the time to target. The time taken by subjects to acquire the target increased significantly as the movement distance increased, for both control modalities (Fig. 5, *p* < 0.05, degrees of freedom = 6, H = 110.15). The time to target increased significantly with respect to control modality (*p* < 0.05, degrees of freedom = 1, H = 506.28, higher for Experiment 1 than for Experiment 2), but did not change significantly with the interaction term (*p* $=$ 3.98, degrees of freedom = 6, H = 6.22). In the second stage, an updated SRH test omitting the interaction term showed that the time to target increased significantly with respect to movement distance (*p* < 0.05) and increased significantly with respect to control modality (*p* < 0.05, higher for Experiment 1 than Experiment 2). A linear regression showed that the time to target increased linearly with respect to movement distance for Experiment 1 (slope = 0.01, $R^2=0.96$) as well as Experiment 2 (slope = 0.01, $R^2=0.53$). These results showed that the peak velocity magnitudes and the time to target scaled with respect to the movement distance for the same control modality, but were significantly different when compared across control modalities.

Seven independent F-tests were conducted to test the difference in variance of peak velocity between the two experiments for the same movement distances. For example, peak velocities measured for a target distance of 50% from Experiment 1 were tested against peak velocities measured for a target distance of 50% from Experiment 2, and so on. All F-tests showed that the variance in peak velocities for all target distances was greater for Experiment 2 than the peak velocities for the corresponding target distances for Experiment 1 (*p* < 0.05, see Fig. 4).

Seven independent F-tests were also conducted to test the difference in variance of time to target between the two experiments at the same movement distances. All F-tests showed that the variance in time to target for all target distances was greater for Experiment 2 than the time to target for the corresponding target distances for Experiment 1 (*p* < 0.05, see Fig. 5).

**Figure 6:**
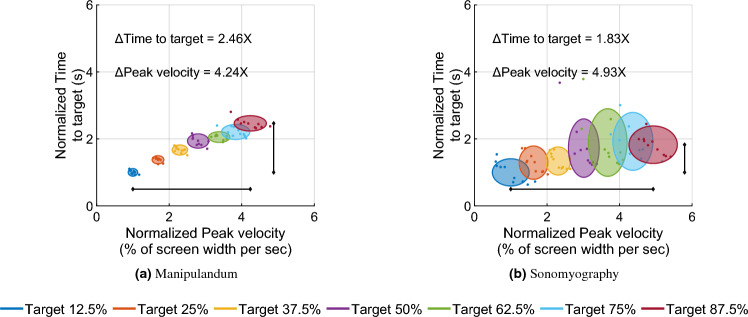
Normalized time to target versus normalized peak velocity. Every trial’s peak velocity and time to target are normalized with respect to the average peak velocity and average time to target for the smallest movement distance. Each color represents a different movement amplitude. The ellipse is centered at the normalized mean of the distribution and the size of the major/minor axis represents the standard deviation along that axis. As the movement amplitude increases (from blue to magenta), the time to target (vertical location of each ellipse) does not change as much as the peak velocity (horizontal location of each ellipse).

**Figure 7:**
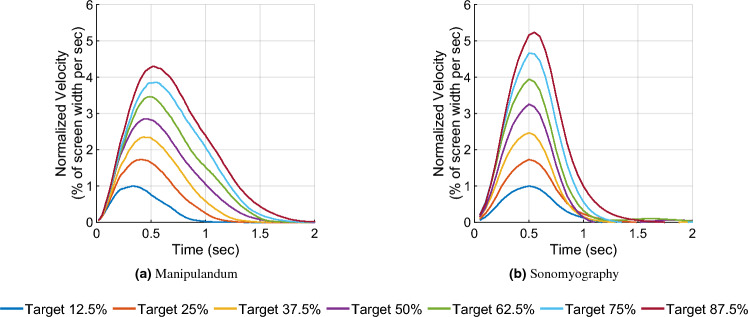
Normalized mean velocity profiles across all subjects versus time. The data is grouped by movement amplitude (each color represents a different movement distance). The peak velocity achieved by the subjects increases as the target moves further away from the start location.

**Figure 8:**
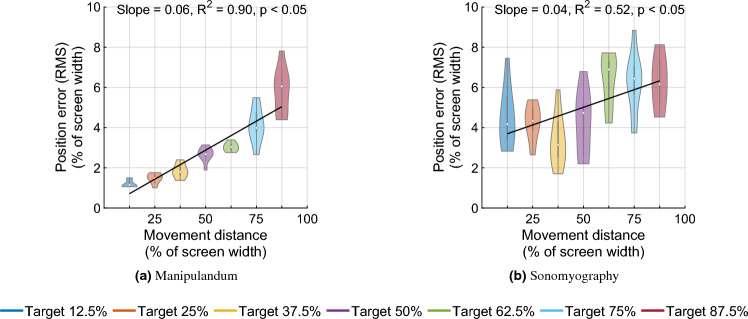
Root mean squared position error between the users’ cursor position and the ideal minimum jerk trajectory. The error increased as the movement distance to that target increased.

**Figure 9:**
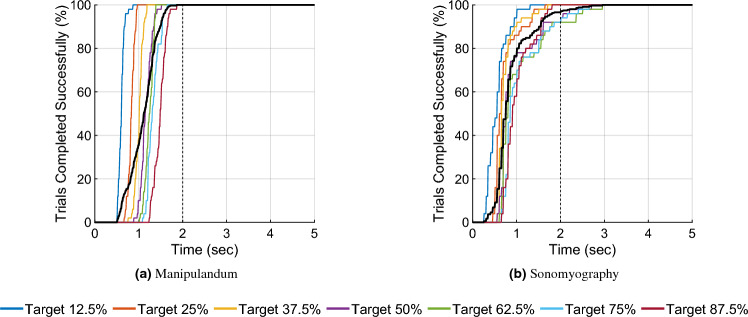
Percentage of trials completed versus time. The data is grouped by movement distance. The percentage of trials completed within a certain time period was inversely proportional to the movement distance. Each color shows a different movement distance, whereas the black line shows the average number of trials across all movement distances. These figures also show that almost all the trials were completed within 2 s.

**Figure 10:**
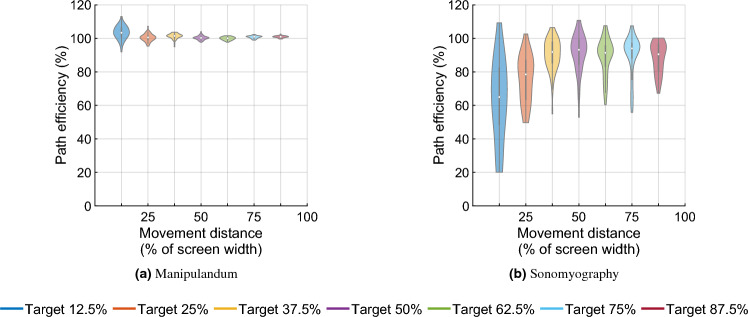
Path efficiency for both control modalities versus movement distance. Path efficiency is the ratio of actual path length to the ideal path length.

Each trial’s peak velocity was plotted against its time to target, normalized with respect to the peak velocity and time to target for the trial’s movement distance (see Fig. 6). Then ellipses were drawn such that the size of the axes corresponded with the standard deviation of peak velocities and time to target respectively, and the center of the ellipse was at coordinates given by the normalized time to target and normalized peak velocity respectively. The normalized peak velocity (normalized to the average peak velocity for the smallest target distance) increased to 4.24 times for the manipulandum and 4.93 times for sonomyography (from the shortest to the longest movement distance). However, the normalized time to target (normalized to the average time to target for the smallest movement distance) increased to only 2.46 times for the manipulandum and 1.83 times for sonomyography (from the shortest to the longest movement distance. For both control modalities, the relative difference in peak velocity (difference in the horizontal location) was more than the relative difference in time to target (difference in the vertical location).

Mean velocity traces were plotted against time to show scaling of peak velocities on average for every target, as well as the systematic shift in time to achieve peak velocity, which is a feature of the minimum jerk trajectory model (see Fig. 7). For Experiment 1, the time taken to achieve the peak velocity for the farthest target increased by 1.69 times compared to the smallest target, while it increased 0.97 times for Experiment 2.

In the first stage, a two-way Scheirer–Ray–Hare test was conducted to test the effect of control modality and movement distance on the time taken to achieve velocity peak. It increased significantly with respect to movement distance, for both control modalities (Fig. 8, *p* < 0.05, degrees of freedom = 6, H = 26.16). The time taken to achieve peak velocity also increased significantly with respect to control modality (*p* < 0.05, degrees of freedom = 1, H = 518.56, higher for Experiment 2 than Experiment 1), and also changed significantly with the interaction term (*p* < 0.05, degrees of freedom = 6, H = 26.18). Since the interaction effect was significant for time to velocity peak, in the second stage, two separate Kruskall–Wallis (KW) tests are conducted for subsets of the trials. The first subset consists of trials performed at the seven movement distances corresponding to the manipulandum modality, and the second subset includes the remaining trials performed at the seven movement distances obtained via sonomyography. After subsetting, note that there is just a single factor (movement distance) for each KW test. The test results indicated that movement distance is statistically significant for both levels of control modality (*p* < 0.05). We elected to fix control modality for the Kruskal Wallis tests to minimize the total number of tests.

To assess the performance of subjects, the trajectory of each trial was compared to the minimum jerk trajectory. For every target position, we computed the average time to target and used this time to compute the minimum jerk trajectory for that target position. The time series root mean square error was computed between the average position trace and the minimum jerk trajectory, and termed as position error. In the first stage, a two-way Scheirer–Ray–Hare test was conducted to test the effect of control modality and movement distance on the position error. It increased significantly with respect to movement distance, for both control modalities (Fig. 8, *p* < 0.05, degrees of freedom = 6, H = 59.74). The position error increased significantly with respect to control modality (*p* < 0.05, degrees of freedom = 1, H = 37.11, higher for Experiment 2 than Experiment 1), and also changed significantly with the interaction term (*p* < 0.05, degrees of freedom = 6, H = 24.002, higher for Experiment 2 than Experiment 1). Since the interaction effect was significant for position error, in the second stage, two separate Kruskall–Wallis (KW) tests are conducted for subsets of the trials. The first subset consists of trials performed at the seven movement distances corresponding to the manipulandum modality, and the second subset includes the remaining trials performed at the seven movement distances obtained via sonomyography. After subsetting, note that there is just a single factor (movement distance) for each KW test. The test results indicated that movement distance is statistically significant for both levels of control modality (*p* < 0.05).

A linear regression showed that the position error increased linearly with respect to movement distance for Experiment 1 ($R^2=0.90$) as well as Experiment 2 ($R^2=0.52$). The position error was not predominantly negative or positive, meaning on average, the user trajectory was not strictly above or below the minimum jerk trajectory. For Experiment 1, the average root mean square error between the users’ cursor position and the minimum jerk trajectory was between 1.2 and 5.78% of the screen width, increasing with target position. For Experiment 2, it was between 4.45 and 6.22% of the screen width, also increasing with target distance. All the position and velocity traces were tightly packed with the standard deviation across trials ranging from 0.53 to 2.36% for Experiment 1, and 4.27–5.82% for Experiment 2, both increasing with target distance. These results show that both control modalities had low variation across subjects.

All the trials for Experiment 1 , and 96.57% of the trials for Experiment 2 were completed within 2 s (see Fig. 9). The average path efficiency was $101.03\% \pm 2.2\%$ for Experiment 1 and $84.06\% \pm 16.81\%$ for Experiment 2. Seven independent F-tests were conducted to test the difference in variance path efficiencies between the two experiments at the same movement distances. All F-tests showed that the variance in path efficiencies for all target distances was greater for Experiment 2 than the variance in path efficiencies for the corresponding target distances for Experiment 1 (*p* < 0.05, see Fig. 10). The ideal path length was defined as the distance between the starting position and the center of the target position. However, during certain trials, subjects seem to have stopped moving after achieving the outer edge/radius of the target. This meant that the target was ”achieved”, but with a path length shorter than the ideal path length, making some path efficiencies greater than 100%.

## Discussion

In this work, we used sonomyography to investigate the time course of control signals derived from imaging forearm musculature during a virtual target acquisition task, and compared it to control signals derived during cursor movements based on physical arm reaching movements. For both control modalities, the peak velocity and the time to target increased linearly with respect to movement distance. The change in time to target for the different movement distances was smaller for control signals derived from sonomyography than for the control signal derived from the manipulandum. Once the target was acquired, there was a greater deceleration phase for the manipulandum than for sonomyography (Fig. 7). The position traces of the cursor controlled by both modalities exhibited a trajectory close to the minimum jerk trajectory (Fig. 2), and the velocity profiles had a single bell-shaped peak at approximately the movement mid-point (Fig. 3). These results were consistent across subjects and across trials within each subject. Thus, the sonomyography-based movement trajectories derived from the users’ forearm muscles during wrist flexion/extension were consistent with behavior previously documented for coordinated multi-joint movements of the upper limb. This is novel since we report the existence of the minimum jerk trajectories using only internal measurement of muscle activation (sonomyography), rather than kinematic or external tracking of the effector (i.e. accelerometer on the arm).

### Characterizing movement quality in control tasks

The virtual cursor control task is often used to evaluate the performance of human machine interfaces^70–75^. Traditionally, surface electromyography has been used to decode motor intent and drive the cursor during a virtual target achievement control task^76–80^. Various algorithms have been used to generate the control signal, including pattern recognition, linear regression, etc, and the derived signal can be used to control either the velocity or position of the cursor. The performance of the control paradigm is characterized using standard metrics such as movement time, path length, path efficiency, completion rate, accuracy, precision, etc.^81^. However, these metrics describe user performance without characterizing the time-course of the signal i.e. the evolution of the signal with respect to time. Therefore, with these standard metrics it is not possible to evaluate the movement quality in terms of its similarity to naturalistic human movements.

### The minimum jerk trajectory during human movement

Hogan^82^ proposed that the central nervous system reflects the minimum jerk trajectory during the path-planning stage when a target is pre-selected. Others^56,64,83–87^ have shown that similar behavior is displayed during a variety of tasks such as arm reaching^55–57^, catching^58^, drawing movements^59^, vertical arm movements^60^, head movements^61^, saccadic eye movements^62^, chewing^63^, and several other motor skills^64^. Highly coordinated multi-joint upper limb movement has been shown to exhibit minimum jerk trajectories^55,56,60,88^, but to the best of our knowledge, these results have not been shown with respect to movement trajectories derived directly from imaging the musculature controlling a single joint. That is, the majority of the previous studies have documented the minimum jerk trajectory by tracking the kinematics of the moving body part, but not shown this to hold true when the control was derived using an internal measurement like ultrasound imaging of forearm muscles. Similar to the prior work described above, here we demonstrated that virtual end-point trajectories derived from sonomyography also follow a minimum jerk trajectory.

Typical point to point movement trajectories show a scaling of peak velocities and time to acquire targets based on target distance^89,90^. Neurologically healthy subjects follow typical movement trajectories based on distance to the target, and have very minimal abrupt changes due to error correction as they move closer to the target. During point to point reaching tasks, as the movement amplitude increases, the peak velocity (height of the bell-shaped velocity curve) increases proportionally. We have shown this behavior in the sonomyography trajectories as well (Fig. 3b). In prior work^54^ we have demonstrated that sonomyographic signals are proportional to motion completion level and here we offer evidence that the minimum jerk trajectory is followed at the single joint level. These two results suggest that it may be possible to use sonomyography to investigate how other motor control policies based on muscle activation apply at the single joint level. For example, sonomyography could also be used to investigate the properties of motor control at the single joint level in individuals with a limb deficiency or movement disorders.

Common motor control principles have been found to hold for various tasks under many different environmental and task performance conditions^55–64,91^. However, there is some debate over whether the typical motor behaviors are the result of internal properties of the motor system or they are simply the result of responses to visual and perceptual information. For certain periodic bimanual tasks, it has been argued that the motor system is subordinate to the visual/perceptual constraints while performing visuospatial motor tasks^92^. In the current work, users had complete access to visual feedback, and we did not perturb the visual representation of the task in real-time during the task. However, we have tested the effect of removing visual feedback of the cursor on task performance (errors, time to target, etc)^93^. In future work, we plan to test the effect of removing visual feedback on the dynamics of the performance reported here.

### Applications in rehabilitation engineering

Our results demonstrate that it is possible to achieve naturalistic control using control signals derived from muscle. Sonomyography is an emerging modality that is being used for controlling upper and lower limb prosthetic devices^94^, but can also be used to control biomechatronic devices such as exoskeletons^95–97^ and prosthetic hands^18,54,93^.The results presented in this work have direct relevance to designing control strategies for such devices, that reflect the natural movements of the human body. Surface electromyography has been the predominant method of decoding motor intent in persons with movement disabilities using electrical signals from the surface of the skin^26^. This has proved to be a valuable tool, but it faces some challenges in terms of low signal to noise ratio and specificity due to the measurements being made at the surface level. However, techniques based on ultrasound imaging track the spatiotemporal patterns of deep lying musculature, giving access to information beyond surface level measurements.

### Other applications of positional control

Sonomyography, as well as other modalities that enable robust positional control, could also be used to closely examine feedforward and feedback control mechanisms during grasping movements. These mechanisms are efficiently combined during grasping tasks^98–103^. The action usually starts with feedforward control until some form of haptic feedback is available from the environment. When such haptic feedback is available, there is a trade-off between energy efficiency and slip prevention, that allows the user to maintain a force that is higher than the minimum necessary force for just grasping the object^104^. Users often exhibit higher grip force while using only a feedforward control strategy in the absence of visual feedback^105,106^. Prosthetic users rely heavily on feedforward mechanisms^107^ as visual information is often the only type of feedback available to them. These control paradigms could be further studied using sonomyography, by comparing muscle deformation following onset of movement but before object interaction, with muscle deformation after object interaction has taken place. The magnitude and timing of these changes could be studied alongside electrical measurements preceding the muscle activation, to further inform our understanding of how the motor systems directs object manipulation in the real world. The current results show that it is possible to study the time course of such interactions and characterize how the movement quality is affected after the onset of a movement disorder or sensory loss. Several studies have shown that adaptation to errors induced by external forces is very natural for able bodied individuals^108–112^, but this mechanism is affected in persons with neuromuscular disorders^113–117^. Sonomyography could be another tool to study motor learning and adaptation by characterizing the time course of muscle movement as a reaction to external forces during certain tasks.

In our current study, the sonomyographic control signals did not directly track individual muscle boundaries or landmarks in the musculature. We aim to develop more advanced signal extraction techniques that will track individual muscle compartments in the future. In addition, the current studies were conducted on able bodied subjects. It would be very informative to investigate how these results compare to the movement trajectories exhibited by persons with limb deficiency as well as other neuromusculoskeletal impairments under the same protocol. In this work, although we placed the ultrasound transducer in an approximately similar spot for all participants as described in the methods section, we did not standardize the probe placement perfectly. Since our signal extraction algorithm is trained separately for every user, this was not a huge problem, but if we move towards tracking physical muscle/bone landmarks then we will also need to standardize probe placement. In this study, we showed all the results using only one hand motion (wrist flexion/extension). However, in our previous work^18,54^ that the sonomyography signal tracks motion completion level accurately for a range of hand motions. That is why, we believe that we could explore similar trajectory tracking using other motions in future work as well. Additionally, there is a slight difference in how the targets were presented for the two experiments, in terms of the mirroring of the hand used for control. For Experiment 2, we do not believe that this mirroring had a significant effect on the results, as the sonomyography signal has been shown previously to have an average stability error of 6.51% for able bodied subjects and persons with amputation. Our current study was not powered to evaluate differences in limb dominance. Future studies could investigate the impact of limb dominance on control trajectory performance.

## Conclusions

We have shown in this work that (1) sonomyography is a tool capable of investigating the time course of muscle deformation when the users engage in isotonic movements, and (2) subjects demonstrate comparable planning and execution of a virtual cursor control task using a hand-held manipulandum or imaging of the forearm muscles. Movement trajectories based on isotonic activation of the limb muscles sensed through sonomyography, and those resulting from arm movement show similar characteristics: single bell-shaped velocity profiles, scaling of peak velocity based on movement distance, shift in time to achieving peak velocity, and increase in time to target based on movement distance. These results have been shown previously with respect to kinematics of whole limb movements, but we have shown that the same control relationships are reflected in control signals derived at the muscle level. In the future, these findings could enable the use of sonomyography and other robust position control signals to study the extent to which motor control relationships are preserved in individuals with neuromusculoskeletal impairments, and how these relationships are affected by multi sensory feedback modalities.

## Data Availability

The datasets generated during and/or analyzed during the current study are available from the corresponding author on reasonable request.
